# Negative Affect, Type D Personality, Quality of Life, and Dysfunctional Outcomes of Total Knee Arthroplasty

**DOI:** 10.1155/2019/6393101

**Published:** 2019-01-03

**Authors:** Matthias Vogel, Christian Riediger, Martin Krippl, Jörg Frommer, Christoph Lohmann, Sebastian Illiger

**Affiliations:** ^1^Universitätsklinik für Psychosomatische Medizin und Psychotherapie der Otto-von-Guericke-Universität Magdeburg, Leipziger Straße 44, 39120 Magdeburg, Germany; ^2^Institut für Psychologie der Otto-von-Guericke-Universität Magdeburg, Universitätsplatz 2, Geb. 24, 39106 Magdeburg, Germany; ^3^Universitätsklinik für Orthopädie der Otto-von-Guericke-Universität Magdeburg, Leipziger Straße 44, 39120 Magdeburg, Germany

## Abstract

**Background:**

Type D personality (TDP) is a sign of tapered stress and compromises treatment outcomes including those of hip arthroplasty. The common dissatisfaction with total knee arthroplasty (TKA) is predicted by fear avoidance, pain catastrophizing and emotional lability, with poor quality of life (QoL) reflecting these strains. This study is the first to investigate the influence of TDP on TKA assuming (1) negative affect (NA) to be linked to fear avoidance and to increased dissatisfaction with TKA and (2) the expression of NA and social inhibition (SI) to not be stable over time.

**Method:**

We studied 79 participants using the brief symptom inventory-18, the pain-catastrophizing scale, the Tampa scale of kinesiophobia, the SF-36, and the WOMAC preoperatively and 12 months postoperatively. *T*-test and regression were used to compare the variables of interest between groups built based upon outcome severity.

**Result:**

NA at follow-up predicted knee pain (*p*=0.02) and knee function (*p* < 0.01) at follow-up. Contrarily, increased expressions of NA/SI at follow-up were predicted by NA (*p*=0.04) and rumination (*p*=0.05) at the baseline.

**Conclusion:**

The present results suggest the postoperative increase of NA to be linked to dysfunctional outcomes of TKA due to an interaction with pain catastrophizing. Baseline self-rated physical health did not connect to the dissatisfaction with TKA 1-year postoperatively.

## 1. Introduction

Chronic pain is a widespread medical problem of an equifinal character usually involving psychological, psychosomatic, and psychosocial factors [1–3]. The fear avoidance model of chronic pain is a widely accepted and influential psychological model for the explanation of chronic pain [4]. It posits that pain catastrophizing and kinesiophobia (i.e., the fear of movement and re-injury) constitute a magnifying focus of attention directed at somatosensory perceptions, thus inflating this perception in terms of a pain-related exaggeration of fearful anticipation of the pain getting worse and worse. In addition, the fear avoidance model also views psychosomatic and psychiatric disorders, such as depression or panic disorder, as conditions usually enhancing this zoom on pain signals [5]. Osteoarthritis (OA), a degenerative joint disease, serves well as an example of the progressive, complex, and multifaceted nature of chronic pain [6]. Regarding TKA, which is an ultima-ratio therapeutic option for the chronic painful condition created by OA of the knee, its results are compromised by pain catastrophizing, depression, and other psychopathology [7, 8]. This effect is obviously based on the underlying association between chronic pain and emotional vulnerability determined at a personality level, which is being reflected by the reported associations between neuroticism and borderline criteria on one hand and worse outcomes of TKA on the other [9, 10]. Type D personality (TDP), introduced by Dennollet et al. [11], refers to a general inclination to psychological distress, with the main source of that distress being the components of TDP, negative affect (NA), and social inhibition (SI). Denollet [12] depicts TDP as a personality configuration predisposing to reticence, distress, and conscious suppression of emotions. NA is described as depression, dysphoria, anxiety, hostility, anger, or irritability [13, 14]. SI, on the contrary, is understood as a tendency to not express these negative emotions to others [14]. The synergism between these two personality characteristics is deemed to maximize the experience of chronic stress in those affected [14, 15]. Therefore, TDP does not necessarily surprise by its link to chronic stress-mediated medical conditions such as cardiovascular disease [14, 16]. Lambertus et al. [17] have recently shown that there is a marked comorbidity of TDP with psychiatric disorders, mainly social phobia, dysthymia, and personality disorders (e.g., avoidant or compulsive). TDP is also a risk factor regarding total hip arthroplasty (THA) [18], while it has not yet been investigated with regard to arthroplasty of the knee. However, certain findings regarding TDP, other psychopathology, or its underlying cognitive and emotional processes suggest a meaning of TDP also in knee OA and related arthroplasty. Wong et al. [9] have reported NA to foster catastrophic thinking, including the catastrophic attributions to pain. Likewise, Leeuw et al. [19] opine the pain threshold to be lowered by NA, and Vlaeyen and Crombez [20] argue alike with regard to disability in the context of chronic pain. Pain catastrophizing, in turn, is an important predictor of the postoperative algofunction [7] which raises interest in exploring its interplay with NA and SI in the context of TKA. Along these lines, negative affectivity is linked to chronic pain [21] with this relationship being reflected by a link between TDP and (worse) outcomes of hip arthroplasty [18]. Not least, the frequent coincidence of anxiety, depression, and osteoarthritis led [22] to refer to this constellation as a “triple whammy” suggesting it to represent one entity altogether rather than one entity plus comorbidities. Hence, negative affectivity is apparently linked not only to outcomes of total hip arthroplasty but also to OA itself. Yet, no study so far has addressed TDP in the context of TKA.

Given the reported association between TDP and catastrophizing, we speculate that it extends to pain catastrophizing lending an explanation for the association between chronic pain and TDP. Moreover, we speculate, based on prior research, this effect to be mainly accounted for by NA [9]. On the contrary, social inhibition, by its very nature, predisposes to finding oneself alone with one's problems, making less use of social support and establishing less quality of life [23, 24]. Hence, we are expecting a more detrimental effect of SI on QoL. Regarding the course of TDP, we expect varying expressions of it, based upon several studies reporting a subgroup, in whom TDP is not stable. Suchlike was found in patients with myocardial infarction [25], cardiac disease [26], and dialysis [27]. Regarding the expected instability of TDP over time, we also wonder whether it may be an expression of maladaptive coping strategies rather than their cause. At any rate, TDP is linked to lowered quality of life in a variety of diseases, e.g., multiple sclerosis [28], coronary artery disease [29], diabetes mellitus [30], and rheumatoid arthritis [31]. NA is held responsible for effectuating the loss in QoL [31]. Therefore, an increase in the features of TDP, and especially in NA, is likely linked to a decline of quality of life (QoL), based on the literature in the field.

## 2. Method

Seventy-nine patients scheduled for elective primary TKA for osteoarthritis were included in the present study. 72% of the patients lived with a partner, 48% had attended junior high school or have had higher education, 8% of the patients had no training qualification, 13.33% had attended university, and 65.3% had made an apprenticeship, while 4% were unemployed, 56% received a pension, and 32% worked on a regular base. Written informed consent was obtained from all the patients. The study was approved by the local institutional review board. Participants were asked to fill in questionnaires assessing the variables of interest of psychopathological distress, type D features, kinesiophobia, and pain catastrophizing, as well as QoL. This study compares the data collected before the operation and after 1 yr of follow-up. The mean age of the participants was 66.28 (11.26) years. The groups with persistent pain (68.12 + 11.37 vs. 65.07 ± 11.25 yr; *t* = 1.12, *p*=0.3), persistent dysfunction (68.41 ± 11.85 vs. 64.98 + 10.91 yr; *t* = 1.51, *p*=0.1), and reactive TDP (68.22 ± 12.17 vs. 65.07 ± 11.07 yr; *t* = 1.16, *p*=0.3) did not differ from those without regarding age.

### 2.1. Questionnaire Measures

Knee pain and knee function were assessed using the WOMAC pain and function subscales (WOMAC A and WOMAC C). Cronbach's *α* of the WOMAC range from 0.8 to 0.96, and their psychometric properties are judged good [32]. The WOMAC used in this study was the Likert version in the format of a numerical rating scale ranging from 0 to 10.

The brief symptom inventory (BSI-18) [33], a short version of the symptom check list 90, assesses symptoms of depression, anxiety, and somatization in three subscales. Internal consistency for the subscales ranges between 0.79 and 0.91, discriminant and convergent validity are deemed good, and the scale is useful as a screening for psychological distress in physically ill populations.

The pain-catastrophizing scale (PCS) is a 13-item rating scale comprising the subscales rumination (PCS-Rumi), magnification (PCS-Magni), and helplessness (PCS-Help). It assesses thoughts and feelings about pain experience on a 5-point Likert scale. The PCS has proven adequate to excellent internal consistency (Cronbach's *α*: total score: 0.87, PCS-Rumi: 0.87, PCS-Magni: 0.66, and PCS-Help: 0.78) [34].

The Tampa scale of kinesiophobia (TSK) is a thirteen-item rating scale rated on a 4-point Likert scale. Assessing fear of movement and re-injury, it is a valid and reliable instrument with Cronbach's *α* being 0.73 for its German version [35]. The TSK is divided into two subscales termed “activity avoidance (AA)” and “somatic focus (SF).”

The short form 36 (SF-36) assesses eight dimensions of subjective health and two summary scores (the physical and the mental component score, PCSc—this naming shall avoid confusion with the pain-catastrophizing scale, PCS, and the MCS. Reliability, validity, and sensitivity of the SF-36 are deemed excellent also regarding the German version [36]. The SF-36 comprises the dimensions physical functioning, role physical, bodily pain, general health, vitality, social function, role emotional, and mental health and can be summed up using the mental as well as the physical component score.

The outcome variables knee pain (WOMAC A) and knee function (WOMAC C) were dichotomized using the lowest tertile as the cut-off point, allowing for the comparison of the worst third with the remaining group reporting better results. Using severity tertiles is a proven procedure [37] for studying the outcomes of TKA. These groups will be referred to as the persisting pain taxon and the persisting dysfunction taxon. In addition, we used a group variable derived from the follow-up measures of NA and SI. By subtracting the total score of the DS14 after 1 year from the preoperative measure, we arrived at a subset of participants in whom the result had a negative sign. This group is referred to as “reactive TDP,” as the corresponding participants had obviously had experienced an increase of their load of NA/SI during the follow-up. We used *t*-testing to compare continuous variables between groups and *χ*^2^ testing to compare categories. Reported results are understood as two-tailed. Linear as well as binary regressions were then used for the prediction of the outcome variable, at which we used the continuous outcomes knee pain and knee function in linear and the categorical outcome reactive TDP in binary regression, as dependent variables. We selected the independent variables for these procedures according to their significance in the preceding *t*-tests. In addition, we entered the highly interrelated worst function or pain taxon, respectively, as predictors of each other and as a control for mutually shared variance. All regressions were controlled for gender, and no stepwise procedure was applied.

## 3. Results

The following primary outcomes (mean (SD)) need reporting: WOMAC A baseline/follow-up: 5.26 (2.21)/10.94 (10.63); WOMAC C baseline/follow-up: 5.02 (2.30)/44.84 (35.52); NA baseline/follow-up: 8.19 (6.06)/5.34 (4.61); SI baseline/follow-up: 6.87 (4.84)/8.58 (4.18); baseline BSI-total score 6.78 (7.66); baseline PCS total score 17.18 (12.24); and baseline TSK total score 21.05 (6.61). Sociodemographic aspects (partnership, education, and working situation) lacked associations with the persistent pain taxon and with reactive TDP, but there was an association between the persistent dysfunction taxon and not working full-time (*χ*^2^ = 20.84, *p* < 0.01) and between the dysfunctional taxon and not being married as well as living separate from one's partner (*χ*^2^ = 10.34, *p*=0.04). The persisting pain and the persisting dysfunction taxons were highly interrelated (*χ*^2^ = 32.79, *p* < 0.01). The category of type D personality as assessed at baseline was linked to the persisting pain taxon (*χ*^2^ = 4.46, *p*=0.04) but lacked associations with the persistent dysfunction (*χ*^2^ = 0.79, *p*=0.04) and the reactive TDP taxon (*χ*^2^ = 2.28, *p*=0.1).

Those belonging to the persistent pain taxon had worse pain and function also at baseline. Moreover, they showed remarkable psychopathological distress but no elevated scores of pain catastrophizing and kinesiophobia. Members of the persistent dysfunction taxon did not differ from the remaining two-thirds with regard to baseline knee pain and knee function. They did, however, report more pain and negative affectivity at follow-up. Regarding the SF-36, we found a more detrimental effect of the persistent pain taxon on QoL than we observed regarding the persistent dysfunction taxon. On the contrary, those belonging to the reactive type D taxon were not associated with a deterioration of QoL. Tables 1 and 2 show the respective mean values, SDs, and statistics of the group comparisons.

Pain and function at follow-up were best predicted by NA at follow-up. Membership of the reactive type D taxon was best predicted by NA and rumination at baseline. The entire statistics are shown in Tables 3–5. Hence, the outcomes, knee pain and knee function, were interrelated in the present sample but also influenced by NA, which apparently is being reinforced through cognitive processes (e.g., rumination) in reflection of the respective participant's dysfunctional attempt to cope with TKA.

## 4. Discussion

Comparing the more satisfied patients with those unsatisfied 1 year after TKA revealed that the latter had endorsed more psychopathological distress and fear avoidance initially, especially if they belong to the persistent pain taxon. Regarding QoL, the members of the dysfunctional and the persistent pain taxon had reported a decrease mainly with respect to the physical dimensions of QoL. Membership in the persistent pain taxon was best predicted by the persisting dysfunction taxon but also by NA. A surprising finding was the discovery of a reactive TDP taxon in 29 of 75 participants (38.67%) during the 1 yr follow-up, i.e., a group with rising NA/SI levels during the follow-up.

The prevalence of primary TDP at the baseline was lower than that reported for other contexts, e.g., fibromyalgia coinciding with TDP in 57% [38], myocardial infarction coinciding with TDP in 14–25% [23], coronary artery disease coinciding with TDP in 18% [39, 40], heart disease coinciding with TDP in 20–25% [22], and joblessness coinciding with TDP in 53% [41]. In their critical evaluation of the construct of TDP, Coyne and Voogd [42] highlight the notion that scoring high on two correlated measures of stress, NA and SI, is a sign of massively tapered distress. Hence, the prevalence of TDP in any given population may be viewed as a marker of the distress which this population is exposed to. Obviously, an acute illness (e.g., a myocardial infarction) is more stressful than a chronic condition, such as osteoarthritis, and especially, being struck by a sudden threat to life is conceivably linked to extreme levels of emotional and mental distress. Additionally, socioeconomic influences are predictors of TDP [41], underscoring the importance of the social dimensions of health and the necessity of a bio-psychosocial framing of health and illness, especially with regard to chronic pain. Notably, the central processing of social exclusion is partly based on the same neurobiological substrates as the processing of physical pain [43], which may be mirrored by the persistent pain taxon's restriction in their social role function.

Study hypotheses: contrary to our expectations, we did not find TDP related to worse outcomes of TKA, although it has been reported to coincide with worse outcomes after hip arthroplasty [18, 19]. Rather, the present results are suggestive of a dimensional increase in NA/SI to connect to worse outcomes, although Vissers et al. [18] report TDP to be associated with worse outcomes of THA and reduced QoL 3 months postoperatively [19], which may be a point in time too close to the operation for the acute distress to vanish. Pain at 3 months postoperatively would have to be classified as on the edge of becoming chronic, while still reflecting mechanisms of acute pain related to the recent operation [44]. Moreover, the respective research was not occupied with the knee, but the hip. Given the difference between these indications with regard to the joint function and statics involved, one might speculate that the habit of restrictive and relieving posture may lead to functional problems as a result of malposition due to fear of pain and re-injury [3]. Along these lines, White et al. [45] report a large proportion of patients with knee OA to need help with their personal care and routine needs. They also experience a faster decline in gait speed allowing for less participation to be reached as the disorder progresses, compared to OA of the hip [46].

However, kinesiophobia, as assessed by the TSK, was not linked to the algofunctional outcome 1 yr postoperatively, in the present study. In addition, kinesiophobia decreased during the follow-up, but 39% of the participants experienced the opposite regarding the dimensions of TDP. As our study hypothesis regarding the nonstability of TDP was confirmed, the question arose whether changes in TDP during a postoperative follow-up should be considered a sign of TDP being a mode of adaptation to the operation rather than a sign of being a prerequisite of dysfunctional adaptation. In fact, changing loads of TDP symptoms connected to a stressor are not in line with the view of TDP as a stable trait but suggest TDP to reflect a state altered through the stressor. Coyne and de Voogd [42] state that there may be “inflection points for NA and SI” as dimensions, increasing their influence on the outcome under study, and our results suggest a ruminating coping style in connection with the extensive experience of knee pain and knee dysfunction prior to TKA to participate in such a system of outcome modulators. Notably, this effect seems to be attributable to NA, not SI. Indeed, the prediction of the worst function taxon by NA and rumination points at a synergism between the sufferer's cognitive appraisal of her or his painful physical restrictions and a corresponding affective state is ever changing for the worse. This finding is suggestive of a subgroup of patients undergoing TKA whose ruminating habit of coping finally seems to lower the pain threshold via the induction of negative affectivity [21]. SI, on the contrary, lacked an association with pain and dysfunction of the knee in the present study. Though speculatively, we interpret this finding as related to the differential psychological nature of NA and SI. SI reflects an interpersonal function, whereas NA refers to intrapsychic perceptions. Even though interpersonal dimensions may be correlated to physical outcomes in medicine, this connection is presumably indirect and mediated through the affective, i.e., intrapsychic, evaluation of being interpersonally handicapped, which manifests as NA. The importance of NA for the prediction of postoperative maladaptation is additionally highlighted by findings linking it to psychophysical and neurophysiological predictors of pain, such as the reduction of the conditioned pain modulation and facilitated temporal summation (i.e., inhibitory and excitatory pain modulation processes [47]). After all, different avenues of research bear clues for the categorization of patients at risk of persistent pain after TKA, of which clinicians may wish to make use of in order to improve the results of TKA. On this note, the lack of psychological well-being and the corresponding self-concept as highly incapacitated by their osteoarthritis of the knee are also reflected in a decline of self-rated QoL. Of particular importance, at least with the present study in mind, is the remarkable difference with regard to baseline QoL between those with the poorest outcomes and those who fare better after TKA. An obvious question with regard to the dissatisfaction with TKA is whether the above-outlined attitude changes following a surgical procedure. If not so, the respective patients are likely to continue to characterize themselves as socially handicapped, i.e., making less use of social support as a means of coping [48]. At the same time, severe pain along with emotional lability and pain catastrophizing seem to effectuate postoperative maladaptation. Reasons for this are likely to be found within the scope of individual psychological predispositions, e.g., personality characteristics or a history of trauma [10, 49]. Although emotional lability is linked to QoL in the present study in terms of a trend, the latter was not dramatically worse in the reactive TDP taxon. It was, however, connected to higher baseline scores on the mental component score of the SF-36, underscoring the psychosomatic nature of the interaction between TDP features, TKA and QoL. Again, the self-perception as being isolated and lacking social support may be a reaction to TKA, but it likely oftentimes exists prior to TKA. This, notwithstanding any diseased state, results in multidimensional changes affecting not only physical health but also the socioeconomic status and psychological well-being. Hence, one should bear in mind that QoL is a multifinal concept, projecting various changes in the perception of one's body and mind as well as one's social surrounding and capacities in the process of a medical treatment. That is why, Drewett et al. [50] insist that QoL indices do not reflect the specific result of an operation but rather the extent to which an individual is satisfied by the physical, emotional, and social circumstances of her or his life. Meanwhile, QoL is not restricted to being an outcome but it may at any given time reflect changes at psychological, perceptive, and social levels rather than being a correlate of the medical success of a specific medical treatment in the first place. Therefore, a participant failing to benefit from TKA without a medical cause may partly do so because no change in the above-outlined attitude has occurred. Schneider and Braungardt [51] refer to that clinical attributional style, which apparently structures the doctor-patient relationship on the part of clinicians, as medicalization, that is, labelling the causes of illness as medical and making the indication for a medical treatment based thereon, although the respective pathogenic agent would have rightly been classified as psychosomatic or psychosocial.

In conclusion, the present results are indicative of a complex interaction involving rumination, the experience of pain, negative affectivity, and, not least, disability and its subjective impression. While the data do not allow for any causal explanations, they do have clinical implications and a seminal potential. TDP may not be a trait but seems to function as a state which is induced by surgery-related stress. Both, TDP and QoL, are not outcomes of surgery but reflections of the psychosomatic mechanisms involved in coping with TKA. Future research will face the task of identifying psychological markers indicative of a maladaptive potential and of testing psychotherapeutic interventions aiming at improving the patient's emotional, interactional, and adaptive competencies. Rumination and NA may be reasonable candidates for the early detection of maladaptive copers and worthwhile targets for psychotherapeutic interventions. Moreover, the worse the pain before the operation, the greater the expectation of the patient's dissatisfaction with TKA will have to be. This argues clearly for not delaying the operation under the acceptance of the worsening of knee pain, and it calls for a sufficient pain relief especially in the perioperative phase. As to the caveats of the present study, which is the first to report on TDP in TKA and therefore needs replication, its cross-sectional character and the limited sample size need to be mentioned. The sample is not representative of all patients undergoing TKA, and its generalizability is restricted by the lack of a control group.

## Figures and Tables

**Table 1:** *t*-Testing comparing the taxon-derived groups regarding knee pain and knee function (fu) as well as psychometric measures.

	Womac A	Womac A (fu)	Womac C	Womac C (fu)	PCS total	PCS Rumi	PCS-Magni	PCS-Help	TSK tot	TSK-SF	TSK-AA	BSI GSI	BSI som	BSI dep	BSI anx	NA	SI	NA (fu)	Si (fu)
Low pain relief	6.45	23.15	6.18	81.91	19.7	6.96	4.12	8.96	32.12	9.27	13.84	10.11	2.96	4.07	3.07	9.11	7.63	7.15	9.37
	1.68	8.91	2.02	29.02	13.28	4.62	2.77	7.09	7.32	3.35	4.5	9.04	3.24	3.34	2.95	6.03	6.37	5.53	3.44
High pain relief	4.75	4.64	4.53	26.2	16.55	6.12	3.52	7.0	20.12	7.98	12.14	4.78	1.0	2.39	1.41	8.06	6.57	4.48	8.42
	2.14	4.12	2.16	22.07	11.48	4.42	2.68	5.42	5.96	2.77	3.77	5.16	1.62	3.21	1.95	6.03	4.1	3.7	4.41
*t*	3.44	10.22	3.1	8.83	1.08	0.77	0.9	1.23	1.91	1.78	1.74	2.82	2.95	2.36	2.96	0.73	0.78	2.22	0.97
*p*	<0.01	<0.01	<0.01	<0.01	0.3	0.4	0.4	0.2	0.06	0.08	0.09	<0.01	<0.01	<0.01	0.04	0.5	0.4	0.03	0.3
Low fct	6.0	22.67	6.01	90.43	16.0	5.43	3.14	7.43	21.55	8.55	13.0	9.48	2.67	3.67	3.14	9.19	5.95	8.55	10.05
	1.84	10.73	1.85	24.01	11.93	4.18	2.48	6.05	7.29	2.98	4.77	9.5	3.15	3.64	3.15	6.45	5.7	5.35	2.96
High fct	5.02	5.83	4.7	25.46	18.23	6.72	3.85	7.87	20.88	8.35	12.52	5.94	1.4	2.85	1.71	8.06	6.88	4.54	8.33
	2.02	5.59	2.24	17.37	12.05	4.48	2.83	6.0	6.03	2.85	3.76	6.41	2.2	2.4	2.38	5.32	4.07	3.54	4.29
*t*	1.74	6.79	2.25	12.68	0.71	−1.12	−0.99	−0.28	0.4	0.26	0.44	1.8	1.9	0.94	2.08	0.74	−0.76	3.64	1.66
*p*	0.09	<0.01	0.09	<0.01	0.5	0.3	0.3	0.8	0.7	0.8	0.7	0.08	0.06	0.4	0.04	0.4	0.4	<0.01	0.1
TDPr	5.05	14.62	5.33	56.29	13.24	4.41	2.62	6.21	20.82	8.32	12.5	5.45	1.21	2.62	1.66	5.62	4.9	6.48	10.07
	2.15	13.43	2.33	42.49	11.32	4.35	2.0	5.73	6.49	2.79	4.12	5.26	1.73	2.35	1.91	4.91	3.1	5.24	3.54
No TDPr	5.42	8.77	4.8	34.28	19.87	7.51	4.23	8.48	21.41	8.5	12.91	7.85	2.11	3.37	2.37	10.04	8.15	4.76	7.85
	2.26	8.07	2.28	26.41	11.87	4.22	2.79	6.14	6.46	3.09	4.01	8.88	3.09	3.12	3.08	6.02	5.45	4.07	4.23
*t*	−0.69	2.11	0.93	2.76	−2.4	−3.05	−2.67	−1.59	−0.38	−0.25	−0.43	−1.47	−1.59	−1.11	−1.24	−3.47	−2.93	1.59	2.35
*p*	0.5	0.04	0.4	<0.01	0.02	<0.01	<0.01	0.1	0.7	0.8	0.7	0.2	0.1	0.2	0.2	<0.01	<0.01	0.1	0.02

Fu: at follow-up; WOMAC A: knee pain; WOMAC C: knee function; PCS-Magni: subscale magnification of the PCS; PCS-Rumi: subscale rumination of the PCS; PCS-Help: subscale helplessness of the PCS; TSK-SF: subscale somatic focus of the TSK; TSK-AA: subscale activity avoidance of the TSK; BSI-som: subscale somatization of the BSI; BSI-dep: subscale depression of the BSI; BSI-anx: subscale anxiety of the BSI; NA: subscale negative affect of the DS-14; SI: subscale social inhibition of the DS-14.

**Table 2:** *t*-Testing comparing the taxon-derived groups regarding the subscales of the SF-36.

	Physical functioning	Role physical	Bodily pain	General health	Vitality	Social functioning	Role emotional	Mental health	Physical component score	Mental component score
Low pain relief	19.1	15.63	21.92	47.21	41.27	56.5	48.61	61.12	23.79	48.6
	16.15	33.63	16.32	17.98	21.74	29.34	47.12	18.46	8.28	12.35
High pain relief	33.78	30.49	30.85	57.41	54.29	76.3	62.41	68.74	29.25	53.1
	22.47	42.14	16.98	17.1	16.43	23.38	47.46	16.27	8.09	10.53
*t*	−2.9	−1.6	−2.13	−2.34	−2.86	−3.14	−1.16	−1.81	−2.65	−1.59
*p*	<0.01	0.1	0.04	0.02	<0.01	<0.01	0.2	0.08	0.01	0.1
Low fct	16.25	14.47	19.26	51.21	41.08	56.88	61.40	62.8	22.19	51.4
	16.21	32.61	12.99	16.35	22.02	27.05	48.77	20.48	7.68	12.54
High fct	33.64	26.7	31.27	54.89	53.37	74.73	57.78	66.56	29.11	51.69
	22.2	40.13	17.27	17.84	16.6	25.07	47.35	17.74	7.84	11.46
*t*	−3.2	−1.17	−2.72	−0.77	−2.48	−3.0	0.28	−0.75	−3.23	−0.29
*p*	<0.01	0.3	<0.01	0.4	0.02	0.01	0.8	0.5	<0.01	0.9
TDPr	22.86	26.85	27.39	56.32	49.11	70.09	60.71	68.71	26.33	53.34
	21.41	44.36	19.65	18.01	21.69	31.61	48.05	17.15	9.12	11.54
No TDPr	32.91	27.38	28.71	51.25	50.79	69.19	54.76	63.4	28.71	49.38
	22.3	39.0	16.54	18.0	18.32	23.99	47.61	17.71	8.55	11.65
*t*	−1.89	−0.05	−0.3	1.15	−0.35	0.13	0.51	1.24	−1.1	1.38
*p*	0.06	1	0.7	0.3	0.7	0.9	0.6	0.2	0.3	0.2

TDPr: reactive TDP taxon.

**Table 3:** Linear regression, dependent variable: knee pain at follow-up.

Total model: *df* = 13; *F* = 2.76; *p*=0.005; *R*^2^ = 0.42	*B*	Std. error	Beta	*T*	Sig.	CI lower	CI upper
Gender	4.33	2.87	0.19	1.51	0.14	−1.44	10.09
NA (fu)	0.77	0.32	0.32	2.41	0.02	0.13	1.41
BSI-som	0.90	1.09	0.20	0.82	0.41	−1.29	3.09
BSI-dep	−0.51	0.86	−0.13	−0.59	0.56	−2.24	1.23
BSI-anx	−0.94	1.13	−0.22	−0.83	0.41	−3.21	1.33
WOMAC A	0.97	1.21	0.19	0.80	0.43	−1.47	3.41
WOMAC C	0.89	1.34	0.18	0.67	0.51	−1.80	3.59
Physical functioning (SF-36)	0.01	0.12	0.02	0.08	0.94	−0.24	0.26
Bodily pain (SF-36)	0.17	0.14	0.27	1.17	0.25	−0.12	0.45
General health (SF-36)	−0.03	0.09	−0.05	−0.36	0.72	−0.21	0.15
Vitality (SF-36)	−0.02	0.10	−0.03	−0.19	0.85	−0.21	0.17

fu: at follow-up; NA: negative affect; WOMAC A: knee pain; BSI-som: subscale somatization of the BSI; BSI-anx: subscale anxiety of the BSI; BSI-dep: subscale depression of the BSI.

**Table 4:** Linear regression, dependent variable Womac-C (fu).

Total model: *df* = 9; *F* = 4.16; *p* < 0.01; *R*^2^ = 0.43	*B*	SE	β	*T*	*p*	CI lower	CI upper
Gender	4.60	9.40	0.06	0.49	0.63	−14.29	23.49
NA (fu)	3.45	0.99	0.43	3.50	<0.01	1.47	5.44
BSI-anx	−0.37	2.08	−0.03	−0.18	0.86	−4.55	3.81
Physical functioning (SF-36)	−0.32	0.36	−0.18	−0.88	0.38	−1.04	0.41
Bodily pain (SF-36)	−0.23	0.33	−0.11	−0.70	0.49	−0.90	0.44
Vitality (SF-36)	−0.05	0.29	−0.03	−0.18	0.86	−0.63	0.53
Physical component score (SF-36)	−0.60	0.94	−0.14	−0.64	0.53	−2.50	1.29
Employment status	0.84	0.81	0.13	1.04	0.30	−0.78	2.47
Family status	1.65	3.54	0.06	0.47	0.64	−5.46	8.76

BSI-anx: subscale anxiety of the BSI; NA: negative affect.

**Table 5:** Binary regression, dependent variable: reactive TDP taxon.

Cox and Snell *R*^2^: 0.44; Nagelkerke *R*^2^: 0.59	*B*	SE	Wald	df	*p*	Exp(*B*)	CI lower	CI upper
Gender	−0.46	0.65	0.50	1.00	0.48	0.63	0.18	2.25
PCS-SUM	−0.16	0.09	3.10	1.00	0.08	0.85	0.71	1.02
PCS-Magni	0.20	0.24	0.73	1.00	0.39	1.22	0.77	1.95
PCS-Rumi	0.37	0.19	3.72	1.00	0.05	1.45	0.99	2.10
NA	0.14	0.07	4.05	1.00	0.04	1.15	1.00	1.32
SI	0.09	0.08	1.10	1.00	0.29	1.09	0.93	1.29

PCS-sum: total score of the PCS; PCS-Magni: subscale magnification of the PCS; PCS-Rumi: subscale rumination of the PCS; NA: negative affect; SI: social inhibition.

## Data Availability

The SPSS data used to support the findings of this study are available from the corresponding author upon request.
